# *Oryza sativa* ObgC1 Acts as a Key Regulator of DNA Replication and Ribosome Biogenesis in Chloroplast Nucleoids

**DOI:** 10.1186/s12284-021-00498-5

**Published:** 2021-07-12

**Authors:** Ji Chen, Li Wang, Xiaowan Jin, Jian Wan, Lang Zhang, Byoung Il Je, Ke Zhao, Fanlei Kong, Jin Huang, Mengliang Tian

**Affiliations:** 1 College of Agronomy, Sichuan Agricultural University, Chengdu, 611130 China; 2 Division of Applied Life Sciences (BK21+), Graduate School of Gyeongsang National University, Jinju, 660-701 Republic of Korea; 3 College of Ecology and Environment, Chengdu University of Technology, Chengdu, 61005 China; 4 Institute for New Rural Development, Sichuan Agricultural University, Yaan, 625000 China

**Keywords:** Chloroplast nucleoid, cpDNA replication, Obg GTPase, *Oryza sativa*, Ribosome biogenesis, rRNA processing

## Abstract

**Background:**

The Spo0B-associated GTP-binding protein (Obg) GTPase, has diverse and important functions in bacteria, including morphological development, DNA replication and ribosome maturation. Homologs of the *Bacillus subtilis* Obg have been also found in chloroplast of *Oryza sativa*, but their primary roles remain unknown.

**Results:**

We clarify that OsObgC1 is a functional homolog of AtObgC. The mutant *obgc1-d1* exhibited hypersensitivity to the DNA replication inhibitor hydroxyurea. Quantitative PCR results showed that the ratio of chloroplast DNA to nuclear DNA in the mutants was higher than that of the wild-type plants. After DAPI staining, *OsObgC1* mutants showed abnormal nucleoid architectures. The specific punctate staining pattern of OsObgC1-GFP signal suggests that this protein localizes to the chloroplast nucleoids. Furthermore, loss-of-function mutation in *OsObgC1* led to a severe suppression of protein biosynthesis by affecting plastid rRNA processing. It was also demonstrated through rRNA profiling that plastid rRNA processing was decreased in *obgc1-d* mutants, which resulted in impaired ribosome biogenesis. The sucrose density gradient profiles revealed a defective chloroplast ribosome maturation of *obgc1-d1* mutants.

**Conclusion:**

Our findings here indicate that the OsObgC1 retains the evolutionarily biological conserved roles of prokaryotic Obg, which acts as a signaling hub that regulates DNA replication and ribosome biogenesis in chloroplast nucleoids.

**Supplementary Information:**

The online version contains supplementary material available at 10.1186/s12284-021-00498-5.

## Background

In multicellular plants, chloroplasts belong to a family of organelles, the plastids, which share a same set of genome but adopt different functions in different cell types (Lopez-Juez and Pyke 2005). Plastid development from proplastids to photosynthetically active chloroplasts is one of the most important metabolic processes during plant growth (Mandel et al. 1996). Plastid genomes are densely packed into protein-DNA complexes called “plastid nucleoids” (Sakai et al. 2004), and bacterial genomes are similarly organized and called “bacterial nucleoids” (Robinow and Kellenberger 1994). The localization and morphology of plastid nucleoids vary among species and also change in response to developmental stages (Kuroiwa 1991). The nucleoids of proplastid are large, less packed and dispersed along the inner envelope. However, the nucleoids of chloroplasts are small and highly packed, and accommodated in the stroma (Sakamoto et al. 2009). It is not known yet how developmental state affects nucleoid composition and function.

Chloroplast development could be divided into three steps (Mullet 1993; Yoo et al. 2009). The first step involves plastid DNA synthesis, which occurs in the context of chloroplast replication and cell division (Shaver et al. 2008). Plastid genes are transcribed by two distinct RNA polymerases: PEP (plastid-encoded polymerase) is a multisubunit eubacteria-like RNA polymerase whose core subunits are encoded by plastid *rpoA*, *rpoB*, *rpoC1*, and *rpoC2* genes, while NEP (nucleus-encoded polymerase) is a single-subunit phage-type RNA polymerase (Shiina et al. 2005). Plastid genes and operons often contain multiple promoters and can be grouped into three classes, based on the presence of either NEP or PEP promoters or both (Hajdukiewicz et al. 1997). The second step is the chloroplast “build-up” step, which is characterized by the establishment of transcription and translation capacity. At this step, NEP preferentially transcribes plastid genes that encode the elements of transcription and translation apparatus (Hajdukiewicz et al. 1997). Therefore, the transcription and translation activities in the chloroplast are dramatically increased at this stage. In the final third step, PEP exclusively transcribes the chloroplast genes encoding the proteins of the photosynthetic apparatus, which are expressed at high levels (De Santis-MacIossek et al. 1999). Expression of these genes leads to the assembly of photosynthetic apparatus and the enforcement of photosynthetic capacity.

Consistent with the proposed endosymbiotic origin of chloroplasts from ancestral free-living cyanobacteria, chloroplast translation shares many of the features of prokaryotic protein synthesis, for example, the 70S-type ribosomes (Marin-Navarro et al. 2007). Ribosome biogenesis is initiated by transcription of a large pre-rRNA precursors, and they are subsequently processed, folded and assembled with r-proteins (Cheng and Deutscher 2003; Kaczanowska and Ryden-Aulin 2007). These complicated events are also catalyzed and elaborately regulated by diverse non-ribosomal factors (Kaczanowska and Ryden-Aulin 2007). It is noteworthy that among these factors, GTPases play key roles in the events (Karbstein 2007; Koller-Eichhorn et al. 2007). The Obg (Spo0B-associated GTP-binding protein) subfamily of GTPases has been the recent interest in studies for the biogenesis of prokaryotic ribosomes (Maouche et al. 2016; Zielke et al. 2015; Feng et al. 2014). In *Escherichia coli*, overproduction of ObgE allows normal ribosome formation in a cell, but causes defect in a crucial modification of the 50S ribosomal subunit. Moreover, it has been proven that ObgE interacts specifically with L13 (Wout et al. 2004; Jiang et al. 2006). Finally, mutation of *ObgE* affects pre-16S rRNA processing, ribosomal protein levels, and ribosomal protein modification, thereby significantly reducing 70S ribosome levels (Sato et al. 2005). In *Bacillus subtilis,* ribosomal protein L11 is positioned within the ribosome near Obg′s GTPase activating center (Zhang and Haldenwang 2004). These lines of evidence implicate that Obgs function in ribosomal biogenesis as rRNA/ribosomal protein folding chaperones or scaffold proteins, presumably through the binding to rRNAs and/or rRNA-ribosomal protein complexes. However, the depletion of *B. subtilis* Obg causes the increased cell length, abnormal cell shape, and nucleoid condensation (Morimoto et al. 2002). Moreover, *E. coli ObgE* mutant cells have a higher DNA content than wild-type cells, and thus, ObgE was suggested previously to regulate the total DNA content within *E. coli* cells (Dutkiewicz et al. 2002). In another study, it was shown that a basal cellular level of the *Vibrio cholerae* Obg homolog is required to overcome the replication inhibition stress caused by hydroxyurea (HU) treatment (Shah et al. 2008). Strikingly, the predicted chloroplast-targeting Obg (ObgC) is co-purified with the 70S ribosome and other ribosome assembly factors in the plastid nucleoid fraction from maize leaves (Majeran et al. 2012), implying the ObgC may regulate ribosome assembly in plastid nucleoids, where is the site of DNA replication and transcription. From these, it seems to be possible that plant ObgC may play dual roles in DNA replication and ribosome biogenesis.

The plant ObgCs have been found in *Arabidopsis thaliana*, *Oryza sativa* and *Dendrobium officinale* (Bang et al. 2009; Chen et al. 2016; Bang et al. 2012). The plant unique ObgCs are essential for plant vitality, chloroplast development and the biogenesis of chloroplast ribosomes (Bang et al. 2012). Moreover, Arabidopsis AtObgC is prerequisite for the formation of normal thylakoid membranes (Garcia et al. 2010) and the response to the environmental stress signaling (Chen et al. 2014). However, clarification of the evolutionarily conserved functions of diverse plant ObgCs requires further investigation. To demonstrate that eukaryotic ObgC in *O. sativa* has the evolutionarily dual functions are conserved with those of prokaryotic Obgs. *Ds* transposon insertion lines, knockout *obgc1-d1* and knockdown *obgc1-d2* have been identified by RT-PCR analyses (Bang et al. 2012). In this study, we report the molecular characterization of an albino *obgc1-d1* mutant in rice, which encoded a plastid-nucleoid targeted OsObgC1. *obgc1-d1* mutant displayed a hypersensity to DNA replication inhibitor HU. A quantitative examination of cpDNA copy number via quantitative real-time PCR (qRT-PCR) analysis revealed that OsObgC1 regulated the replication of chloroplast DNA. In addition, OsObgC1 controlled plastid protein synthesis via the regulation of rRNA processing and ribosome maturation. We speculate that *O. sativa* ObgC1 acts as a signaling hub that regulates DNA and ribosome biogenesis in the plastid nucleoids.

## Results

### OsObgC1 Is a Functional Homolog of AtObgC

**Fig. 1:**
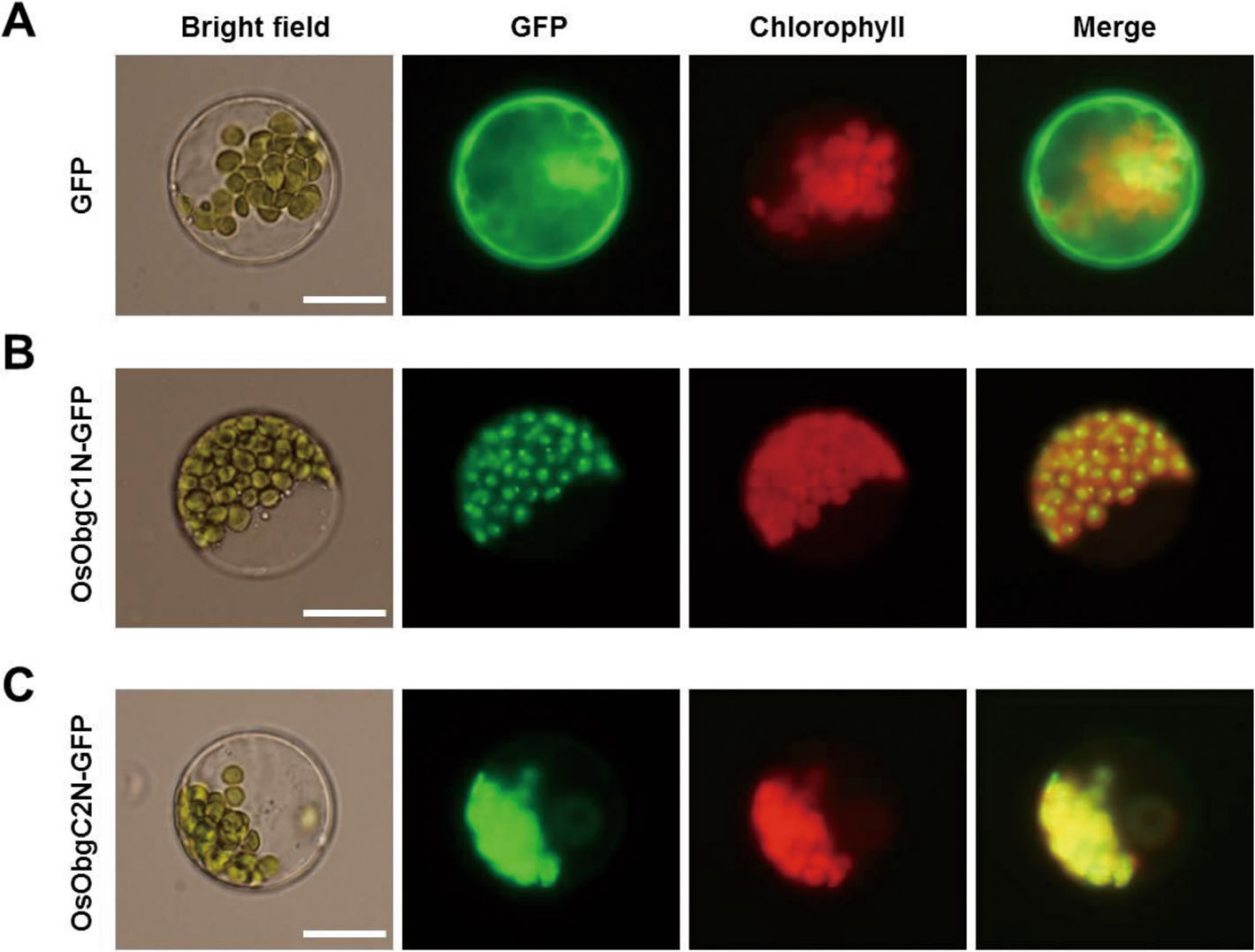
Subcellular localization of rice Obg homologs. Arabidopsis protoplasts isolated from 2-week-old leaves were transformed with GFP (**A**), OsObgC1N-GFP (**B**) or OsObgC2N-GFP (**C**) and were observed with fluorescence microscopy 24 h after transformation. “GFP” represents green fluorescent protein in the transformed protoplasts. “Merge” displays the overlapped image of the GFP and the chlorophyll autofluorescence (Chlorophyll), which was used as a marker for chloroplasts. “Bright” is a bright-field image. White bars in the bright field images represent 20 μm in length

Rice Obg homologs OsObgC1 and OsObgC2, which were predicted as chloroplast-targeting proteins, were identified based on homology searches with *B. subtilis* Obg amino acid sequence (Bang et al. 2009). Primary structure analyses by TargetP (http://www.cbs.dtu.dk/services/TargetP/) and ChloroP (http://www.cbs.dtu.dk/services/ChloroP/) programs revealed that they are predicted to be localized in chloroplasts and have chloroplast transit peptides at their N-termini. To validate the subcellular localization of OsObgC1 and OsObgC2, *OsObgC1N-GFP* and *OsObgC2N-GFP* constructs were transformed into Arabidopsis protoplasts via polyethylene glycol (PEG)-mediated transient expression method. The fluorescence of these gene products was observed under a fluorescence microscopy. As shown in Fig. 1, free GFP was distributed uniformly in the cytoplasm, except chloroplasts, whereas both OsObgC1N-GFP and OsObgC2N-GFP were clearly targeted to chloroplasts. Interestingly, only OsObgC1N-GFP, but not OsObgC2N-GFP, exhibited the punctate staining pattern, just as AtObgC-GFP did (Bang et al. 2009).

**Fig. 2:**
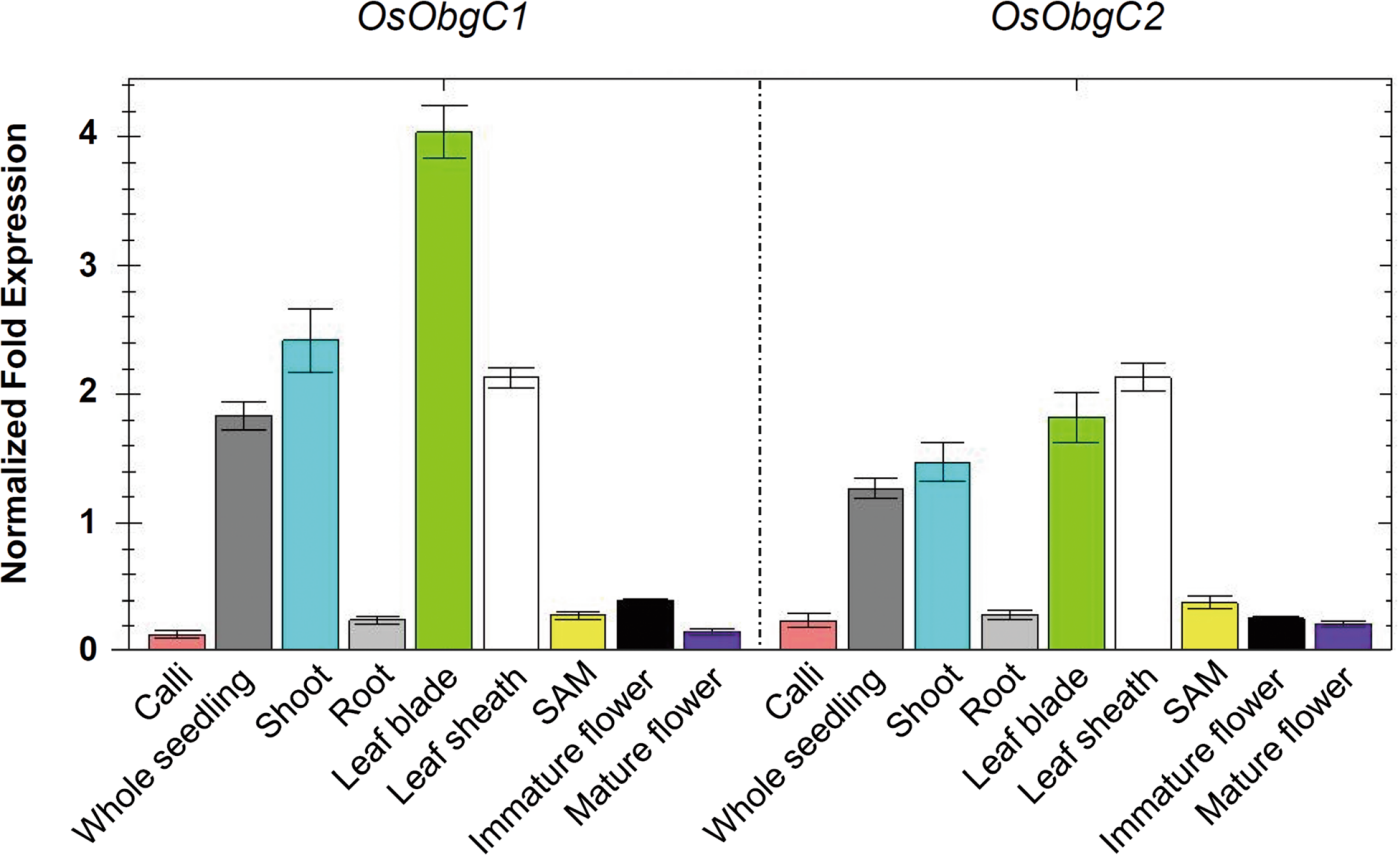
Expression patterns of *OsObgC1* and *OsObgC2* in various tissues. Total RNAs were purified from the following tissues: calli, 10-day-old whole seedlings, shoots, roots, shoot apical meristem (SAM), leaf sheaths, leaf blades, and flowers from rice at reproductive stage. Transcript levels of *OsObgC1* and *OsObgC2* were determined using qRT-PCR with gene-specific primers. Each transcript level was normalized with that of *OsActin* as internal control. Each bar represents the mean ± SD value calculated from three independent experiments

We examined the expression levels of *OsObgC1* and *OsObgC2* in different organs of WT rice by qRT-PCR. Both *OsObgC1* and *OsObgC2* genes were predominantly expressed in green aerial tissues, such as leaves and shoots, and weakly were expressed in shoot apical meristem (SAM), callus, flowers and roots (Fig. 2). This result suggests that *OsObgC1* and *OsObgC2* expressions are in a green tissue-specific manner.

Disruption of the *AtObgC* gene, an essential gene for the biogenesis of plastid ribosome, leads to seed abortion at the early stage of embryogenesis (Bang et al. 2012). To investigate whether OsObgC1 or OsObgC2 may have conserved functions with AtObgC *in planta*, *OsObgC1* and *OsObgC2* genes were respectively overexpressed in Arabidopsis heterozygous *atobgc-t* mutants for complementation analysis. We determined the genotype segregation of heterozygote Arabidopsis *obgc* mutants transformed with *OsObgC1* or *OsObgC2* gene through PCR genotyping (Bang et al. 2009). We obtained survival homozygous *AtObgC* mutants from segregated T1 and T2 plants of transformed heterozygous *atobgc-t* plants with *35S*-*OsObgC1* construct*.* The complemented plants showed normal morphology and seed viability. As shown in Table 1, the overexpression of *OsObgC1* suppressed the embryonic lethality caused by *atobgc-t* mutation, but *OsObgC2* failed to complement *atobgc-t*. The results demonstrated that OsObgC1 is a functional homolog of AtObgC.

**Table 1:** Complementation analyses of Arabidopsis *atobgc-t* phenotype with rice ObgC homologs

Construct		Pop	WT	Hetero	Homo		Total seed no.	Suppression of *atobgc-t*	Chi-squared test
					a	b			
Actual ratio	**Empty vector**	T1	9	10	15		34	No	χ^2^ = 4.484, df = 2, *P* = 0.1063
					0	15			
		T2	72	138	56		266	No	χ^2^ = 2.301, df = 2, *P* = 0.3165
					0	56			
	***35S- OsObgC1***	T1	8	8	5		21	Yes	χ^2^ = 2.048, df = 2, *P* = 0.3592
					5	0			
		T2	58	120	76		254	Yes	χ^2^ = 3.323, df = 2, *P* = 0.1899
					76	0			
	***35S-*** ***OsObgC2***	T1	6	13	9		28	No	χ^2^ = 0.786, df = 2, *P* = 0.6751
					0	9			
		T2	72	129	81		282	No	χ^2^ = 2.617, df = 2, *P* = 0.2702
					0	81			
Theoretical ratio			**1**	**2**	**1**				

Pop designates populations

a is the number of homozygous *atobgc-t* identified by PCR-mediated genotyping

b is the number of aborted seeds

### *obgc1-d1* Mutant Is Hypersensitive to DNA Replication Inhibitor HU

**Fig. 3:**
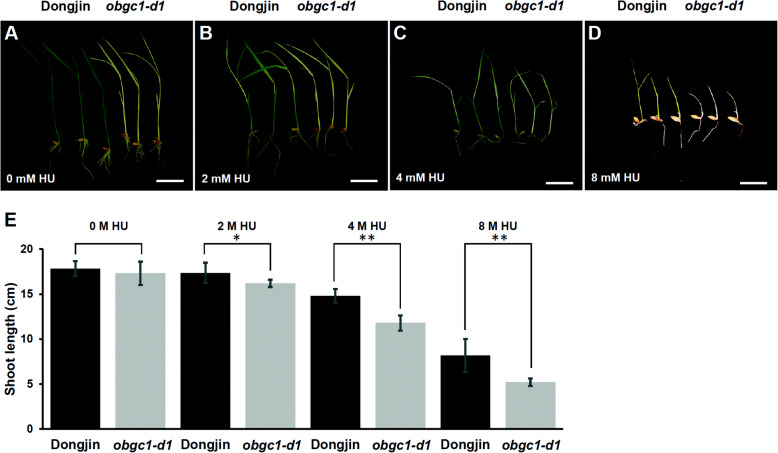
Responses of wild-type and *obgc1-d1* mutant plants to HU exposure. Sensitivity of wild-type (WT) and *obgc1-d1* mutant plants upon exposure to increased concentrations of HU (0, 2, 4, and 8 mM) was determined (**A**-**D**). Shoot length were measured as well (**E**). Seedlings were grown for 10 days in a controlled growth chamber (25 °C, 70% humidity and illumination of 70–80 μmol m^− 2^ s^−1^ white light) with 16 h light and 8 h dark cycle. White scale bars in each growth image represent 4 cm in length. Statistical analysis was conducted by using Student’s t-test. * indicates *p* < 0.5 and ** indicates *p* < 0.1

From recent studies, it was reported that *E. coli obg* mutants are highly sensitive to DNA replication inhibitors that inactivate ribonucleotide reductase (RNR) (Foti et al. 2005; Kint et al. 2012). Although wild-type *E. coli* is tolerant to low concentration of HU, a DNA replication inhibitor, the *obg* mutants are killed due to the formation of unstructured nucleoid (Foti et al. 2005). HU inhibits RNR activity by quenching the tyrosyl radical in the small subunit of RNR, which leads to a reduction in the dNTP pools and stalling of DNA replication forks (Elleingand et al. 1998). To examine the effects of HU on the rate of seedling growth in wild-type and *obgc1-d1* mutant, those plants were grown in HU-containing MS medium. Our results were consistent with the previous report that the retardation of rice growth became more severe as the concentration of HU added was increased (Fig. 3) (Yoo et al. 2009). Wild-type plants displayed a pale green color without growth retardation under a low concentration of HU (2 mM; Fig. 3b), suggesting defects in chloroplast biogenesis. Under the same condition, seedling growth of *obgc1-d1* mutants were slightly delayed (Fig. 3b and e). As the concentration of DNA replication inhibitor (HU) was gradually increased to 4 mM, the mutants were clearly shorter than the wild-type plants as shown in Fig. 3c and e. Finally, under the condition of 8 mM HU addition, plant growth of mutant and wild-type plants were greatly blocked, although those of wild-type plants showed relatively less retardation (Fig. 3d and e). These data suggest that *obgc1-d1* mutant plants are much more sensitive to HU comparing to wild-type plants, implying the involvement of OsObgC1 in chloroplast DNA replication as Obg did in *E. coli*.

### *obgc1-d1* Mutant Displayed a High Copy Number of Chloroplast DNA in both Dark and Light Conditions

In plants, light is an important factor during chloroplast development to regulate chloroplast DNA (cpDNA) replication, the structure of plastid DNA molecules and the instability of cpDNA (Shaver et al. 2008; Zheng et al. 2011). To investigate the effects of light on the cpDNA replication in wild-type and *obgc1-d1* mutant, they were grown in dark and light conditions.

**Fig. 4:**
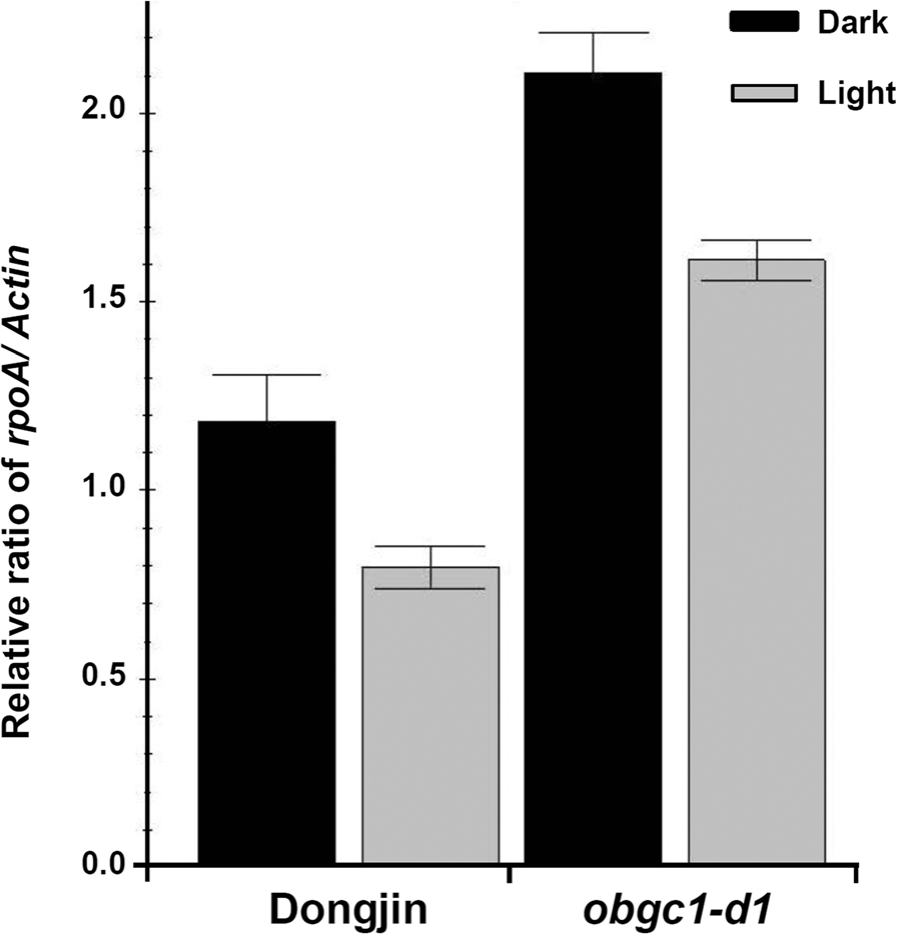
Relative ratios of chloroplast-to-nuclear DNA copy in *obgc1-d1* mutants. The relative ratios of chloroplast DNA (*rpoA*) to nuclear DNA (*Actin*) copies were normalized to the wild-type values. The copy numbers of *rpoA* and *Actin* were determined using qRT-PCR with their gene-specific primers. Black and gray bars indicate the dark- and light-grown plants, respectively. Error bars represent the mean ± SD values calculated from three independent experiments

To check the differences between wild-type and mutant plants in the ratio of copy numbers of chloroplast DNA relative to nuclear DNA, quantitative PCR of *Actin* and *rpoA*, as the representative genes in the rice nuclear and plastid genomes, respectively, was performed. It revealed that the *rpoA*/*Actin* ratios of the mutant plants were much higher than those of wild-type plants in both dark and light conditions (Fig. 4). This indicates that plastid DNA replication is continuously activated by *OsObgC1* mutation, which is reflected as a higher copy number of chloroplast DNA in the *obgc1-d1* mutant plants than those of wild-type plants.

### *obgc1-d1* Mutant Showed an Abnormal Architecture of Plastid Nucleoid

**Fig. 5:**
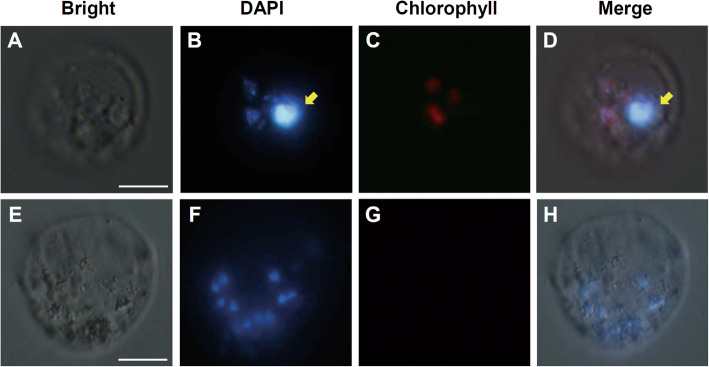
Observation of DAPI-stained plastid nucleoids in the protoplasts isolated from leaves of wild-type and *obgc1-d1* mutant plants. Morphological changes in plastid nucleoids were observed by DAPI staining, using protoplasts isolated from leaves of wild-type (**A**-**D**) and *obgc1-d1* mutant (**E**-**H**) plants. Protoplasts were isolated from leaves of 10-day-old rice seedlings. Signals corresponding to nuclei are indicated by yellow arrows. Bars in the bright images, (**A**) and (**E**), represent 50 μm in length

Previous studies have shown that abnormal nucleoid structure and chromosome-partitioning defect are generated by *E. coli obg* mutation (Foti et al. 2005; Foti et al. 2007). To investigate whether the *obgc1-d1* mutants also exhibit this phenotype, protoplasts isolated from wild-type and *obgc1-d1* shoots were stained with DAPI to label plastid DNA areas (nucleoid) (Fig. 5).

As shown in Fig. 5e-h, the plastids of *obgc1-d1* mutants contained one or a few enlarged nucleoids that had no chlorophyll auto-fluorescence, but had the significantly increased DAPI fluorescence, as those found in proplastids from meristematic cells at leaf bases (Oldenburg and Bendich 2004). By contrast, the chloroplasts of wild-type plants showed that numerous small nucleoids were dispersed in stroma with weak DAPI staining (Fig. 5a-d), indicating that they contained little DNA content. It has been reported that chloroplast DNA is inherited when nucleoids are partitioned into daughter plastids during plastid division (Kuroiwa 1991). Therefore, the number, size and distribution of plastid nucleoid in *obgc1-d1* mutants were clearly distinguished from the pattern of normal chloroplast nucleoid.

### OsObgC1 Is a Chloroplast Nucleoid Localized Protein

Based on the above results, it was proposed that OsObgC1 plays a fundamental role in plastid DNA replication. If OsObgC1 directly affects DNA replication, it should be accommodate in DNA area (nucleoid). Additionally, the spotted and dispersed pattern of DAPI-stained chloroplast nucleoid was reminiscent of punctate staining pattern from ObgCs (AtObgC and OsObgC1).

**Fig. 6:**
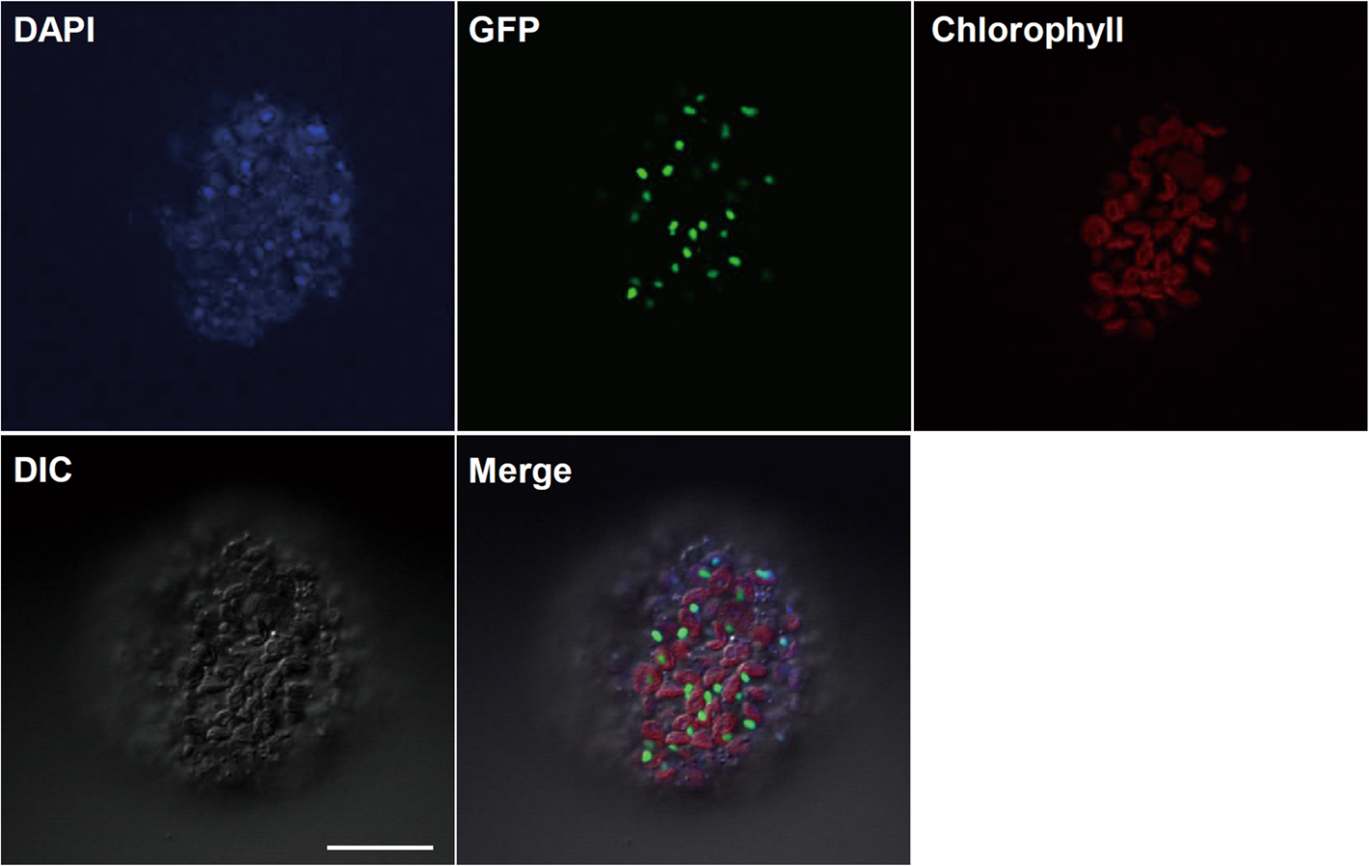
OsObgC1 is localized in the chloroplast nucleoids. Localization of OsObgC1-GFP was examined in tobacco protoplasts by protoplast transient assay. After DAPI staining, DAPI fluorescence, GFP fluorescence and chlorophyll autofluorescence of transformed protoplasts were observed under a confocal microscopy. DIC (differential interference contrast) and Merge (DAPI + GFP + Chlorophyll) images are shown. “Chlorophyll” was used as a marker for to detect chloroplast distribution. The white bar in the DIC image represents 20 μm in length

To look into the location of OsObgC1, by an agrobacterium-mediated transformation method, a construct containing the full-length OsObgC1 attached to GFP under the control of a 35S promoter, was transformed into *Nicotiana benthamiana*. As shown in Fig. 6, GFP-tagged OsObgC1 appeared in small punctate structures throughout chloroplasts (Merge) and were co-localized with DAPI-stained nucleoid DNA (DAPI), suggesting that OsObgC1 is a chloroplast nucleoid-associated protein.

### Loss-of-Function of *ObgC* Influences the Chloroplast-Gene Expression at Transcription and Translation Levels

**Fig. 7:**
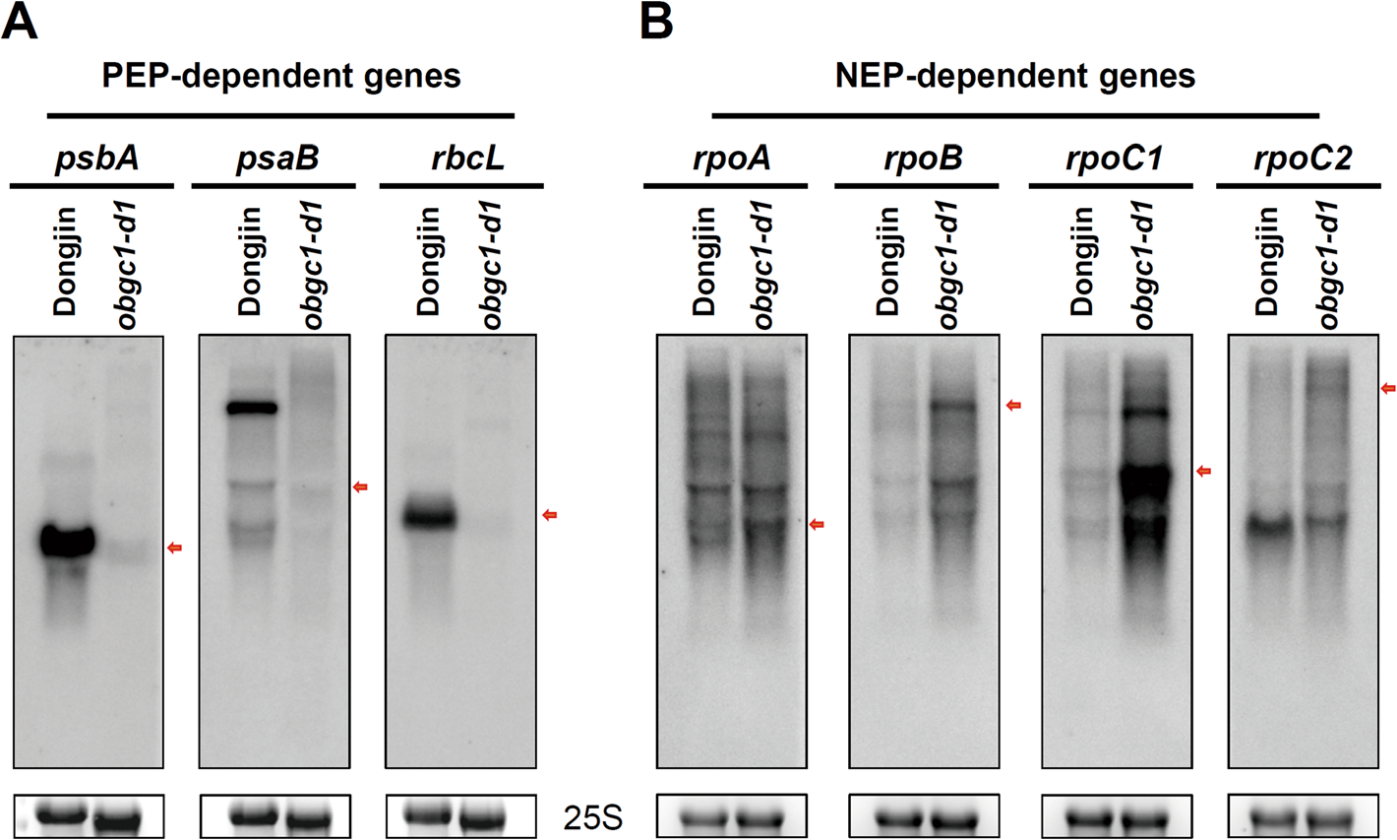
Transcription profiles of chloroplast-encoded genes in rice *obgc1-d1* mutants. Total RNAs (20 μg) extracted from leaves of 10-day-old *obgc1-d1* mutant and wild-type Dongjin seedlings were subjected to northern blot analysis using the indicated gene-specific probes. **A** and **B** indicate the transcription profiles of PEP- and NEP-dependent chloroplast genes in the wild-type and *obgc1-d1* mutant, respectively. The expression levels of 25S were used as the internal control. The mature transcripts of the corresponding genes were indicated with red arrows

Plastid genes have been categorized into three classes as class I, class II and class III, depending on being transcribed by which of two plastid RNA polymerases such as PEP (plastid-encoded RNA polymerase) and NEP (nucleus-encoded RNA polymerase) (Hajdukiewicz et al. 1997): PEP-dependent class I consists of photosynthesis-related genes, such as *psbA, psaB* and *rbcL*; PEP/NEP-dependent class II contains rRNA and several nonphotosynthetic housekeeping genes; and NEP-dependent class III includes *accD*, *ycf2* and the genes (*rpoA*, *rpoB*, *rpoC1* and *rpoC2*) encoding PEP essential subunits. Previously, our macroarray analyses of total plastid genes showed that most of the PEP-dependent class I genes and some class II rRNAs genes were repressed at the transcription level in *AtObgC* RNAi mutants (*atobgc-1*), whereas the up-regulated genes were subjected to NEP-dependent class III (Bang et al. 2012). In *OsObgC1* knockout line (*obgc1-d1*), PEP-dependent genes (*psbA, psaB* and *rbcL*) were severely reduced at transcription levels, whereas the NEP-dependent genes *rpoA*, *rpoB*, *rpoC1* and *rpoC2* were significantly upregulated as shown in Fig. 7. These results suggest that chloroplasts of the *obgc1* mutants contain a defective PEP complex.

**Fig. 8:**
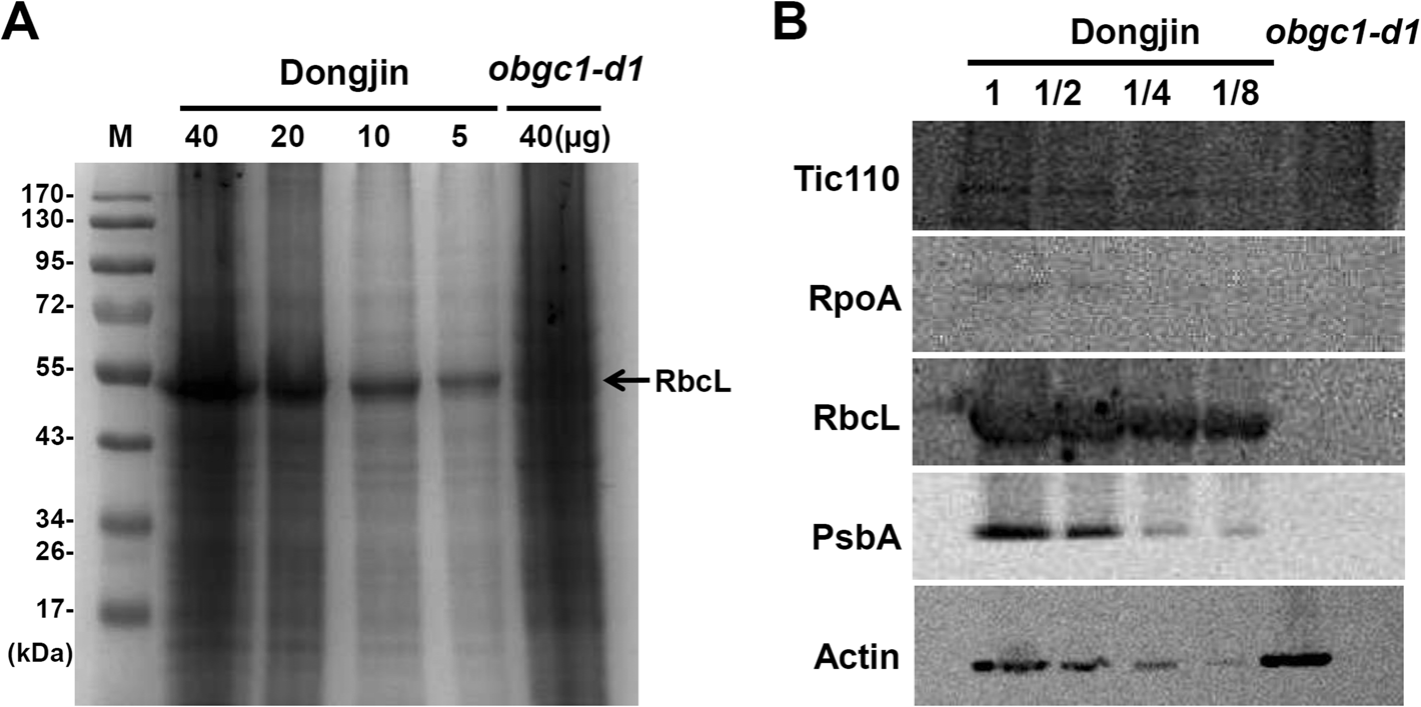
Chloroplast protein accumulation in *obgc1-d1* mutant. Immunoblot analysis compares accumulation of the proteins in *obgc1-d1* and a series of diluted wild-type proteins. **A** Confirmation of gel loading amounts of wild-type and *obgc1-d1*mutant proteins. **B** Total leaf extracts (40 μg of protein or its serial dilution as indicated) were hybridized with the respective antibodies against Tic110, RpoA, RbcL, PsbA and Actin. Tic110 and Actin levels were used as internal controls to normalize the amount of total proteins

To determine whether PEP complexes are indeed disrupted in the rice *obgc1* mutants, western blot analyses were performed using an antibody against RpoA, a component of PEP. As shown in Fig. 7b, *obgc1-d1* mutants showed increased transcription levels of *rpoA*, but interestingly, their protein levels were significantly decreased in these mutants (Fig. 8b). In addition, the synthesis of the chloroplast protein RbcL and PsbA was severely suppressed in mutants, compared with that of wild-type levels (Fig. 8b). These results indicate that synthesis of a wide range of chloroplast-encoded proteins, including the subunits of RuBisCO and photosynthetic system II, were repressed in the mutants. However, translation levels of nuclear-encoded Tic110 and Actin used as negative controls were similar both in the mutant and the wild-type (Fig. 8b). This suggests that the chloroplasts of *obgc1-d1* are defective in protein biosynthesis, leading to insufficient production of PEP. However, as shown in Fig. 7b, *rpoA* transcripts in the *obgc1-d1* mutant were increased as a few PEP-deficient mutants did (Pfalz et al. 2006; Yagi et al. 2012), showing strong up-regulation of typical NEP-dependent genes such as *rpoA, rpoB, rpoC1* and *rpoC2*. According to a previous report by Sugita and Sugiura (1996), it was proposed that *rpoA* was considered as a PEP-dependent gene owing to its presence as a downstream terminal member of the long polycistronic *rpl23* transcription unit (Sugita and Sugiura 1996). However, in many recent PEP-related reports, *rpoA* is mentioned as a NEP-dependent gene (Class III) (Kishine et al. 2004; Yu et al. 2009; Yagi et al. 2012; Pfalz et al. 2006). Based on these, the possibility cannot be excluded that the accumulation of *rpoA* transcript in the mutant may be, at least partly, caused by translational regulation, not by transcriptional regulation.

### *obgc1-d* Mutant Is Defective in Plastid rRNA Accumulation

**Fig. 9:**
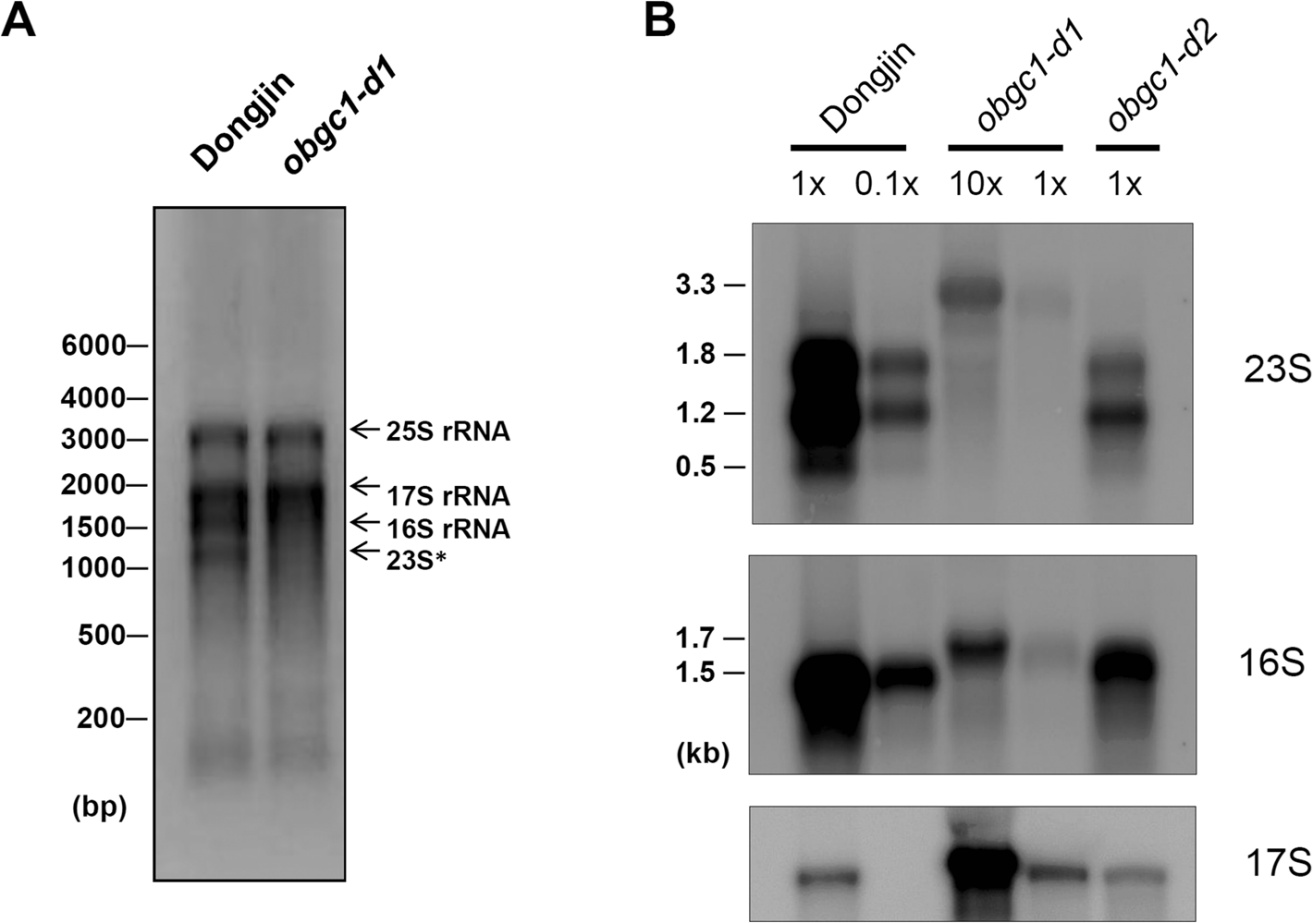
Effect of *obgc1* mutation on the accumulation of plastid precursor rRNA. **A** Abundance of rRNAs. One microgram of total RNAs from 10-day-old wild-type (Dongjin) and *obgc1-d1* seedlings was separated on a denaturing gel, and then stained with EtBr (ethidium bromide). “23S*” (approximate 1.2 kb) is the breakdown product of chloroplast 23S rRNA. **B** *obgc1-d* mutation causes the accumulation of precursor 23S or 16S rRNAs. Northern blot analyses of plastid rRNAs were performed using total RNAs (1x, 1 μg; 0.1x, 0.1 μg; 10x, 10 μg). Blots were probed with gene-specific sequences for 16S/23S rRNAs. Cytosolic 17S rRNA was used as a loading control

In the *obgc1-d* allele, the *Ds* transposants were divided into knockout and knockdown lines, called *obgc1-d1* and *obgc1-d2*, respectively (Supplementary Figure S1). The *obgc1-d2* knockdown line is a result of the somatic transposition of transposon in the *obgc1-d* plant carrying an *Ac* transposase gene. Blots of total RNAs extracted from wild-type or *obgc1-d* rice leaves were probed, to examine whether accumulation, processing or both of various rRNAs were affected by the *obgc1-d* mutation (Fig. 9a). 23S and 16S rRNAs were barely detected in *obgc1-d* mutants, although those were abundant in wild-type plants. However, the cytosolic 17S rRNA bands were shown with equal density in wild-type and mutant plants. This suggests that *obgc1-d* mutation might affect plastid rRNA accumulation (Fig. 9b). Likewise, the mature transcripts of plastid 23S and 16S rRNA were reduced by *AtObgC* mutation (Bang et al. 2012), indicating that the processing of 23S and 16S rRNA and their further maturation were affected in *atobgc-1* mutants.

In the *obgc1-d1* mutant, the levels of plastid rRNA (23S and 16S) transcripts were decreased to less than 1% of wild-type levels (Fig. 9b). After loading of 10-fold amount of *obgc1-d1* mutant RNA, as shown in Fig. 9b, larger transcripts (3.3 kb and 1.7 kb), presumably corresponding to the precursors of 23S and 16S rRNA were detected, respectively. It is interesting that mature 23S or 16S rRNA was undetectable in *obgc1-d1* mutants, while both precursor and mature plastid rRNAs appeared in *obgc1-d2* mutants, as those of *atobgc-1* mutants do (Bang et al. 2009). From this result, it is assumed that changes of *obgc1-d* mutants in plastid rRNA accumulation might depend on the endogenous level of *OsObgC1* transcripts.

### *obgc1-d1* Mutant Fails in Chloroplast rRNA Processing

**Fig. 10:**
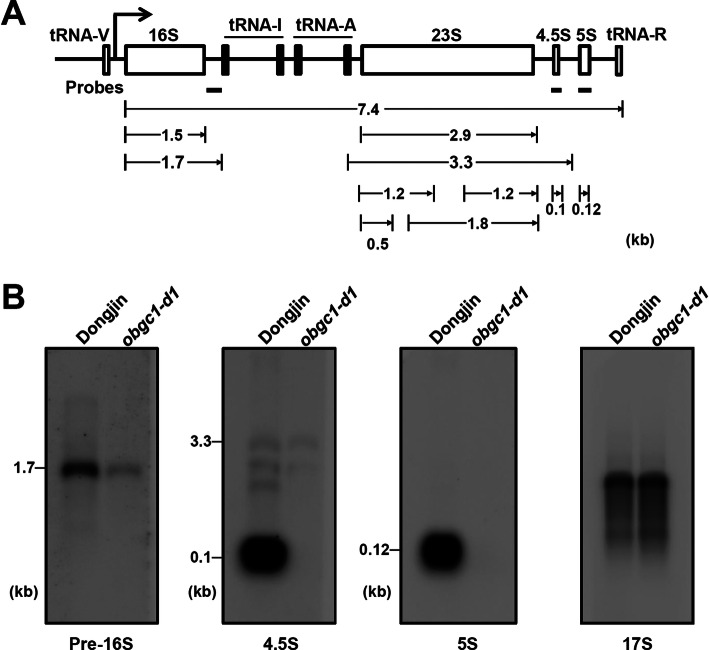
Analysis of plastid rRNA processing in the *obgc1-d1* mutant. **A** A diagram of rice plastid rRNA operon. The probes used in the RNA gel blot analyses in (**B**) are shown as thick black lines under individual rRNA genes. **B** RNA gel blot analysis. One microgram of total RNAs from wild-type (Dongjin) or *obgc1-d1* mutant was loaded and separated in 1.2% agarose-formaldehyde gels, and blots were probed with specific gene sequences to detect processed plastid precursor rRNAs. The cytosolic 17S rRNA shown in the 4th gel image was used as a loading or negative control

A full-length of rice plastid rRNA operon is expressed as a 7.4 kb precursor RNA before both endo- and exonucleolytical processing, which finally generates mature 16S and 5S rRNAs, and 23S–4.5S intermediate is cleaved into mono-cistronic 23S and 4.5S rRNAs in the ribosome (Fig. 10a). Because the larger fragments of mature 23S and 16S rRNA were detected in the *obgc1-d1* mutant as shown in Fig. 9, to positively identify these larger transcripts as the precursors of plastid rRNA, a northern blot was performed again with the probes from precursor specific sites (Bollenbach et al. 2005). Pre-16S probe identified a 1.7 kb band, confirming that this longer transcript (1.7 kb) is the precursor of plastid 16S rRNA (Fig. 10b). When RNA gel blots were analyzed with a 23S-specific probe, various sizes of bands such as 1.8, 1.2 and 0.5 kb in wild-type rice, are appeared in Fig. 9b. It was assumed that there are ′hidden breaks′ during the mature 23S transcript processing, following incorporation into ribosomes (Nishimura et al. 2010; Kishine et al. 2004). Besides these, the 3.3 kb band was assumed as the 23S–4.5S dicistronic precursor RNA. Subsequently, blots were analyzed with a 4.5S probe, the dicistronic 23S–4.5S precursor RNAs and broken products were shown faintly, but 0.1 kb band of the mature 4.5S RNA disappeared in *obgc1-d1* mutants (Fig. 10b). However, in the case of using a 5S probe, neither 0.12 kb band of the mature 5S RNAs nor its precursors (7.4 kb) were undetectable in the mutant. Unlike the other rRNAs, whose precursors and mature forms are highly accumulated, both forms of 5S rRNA accumulate to very low levels (Bollenbach et al. 2005). Since the operon is transcribed under the control of a single promoter, either the precursor and/or mature 5S rRNAs may become unstable caused by the degradation in chloroplasts of the mutant (Sharwood et al. 2011).

### OsObgC1 Plays a Fundamental Role in Plastid Ribosome Assembly

**Fig. 11:**
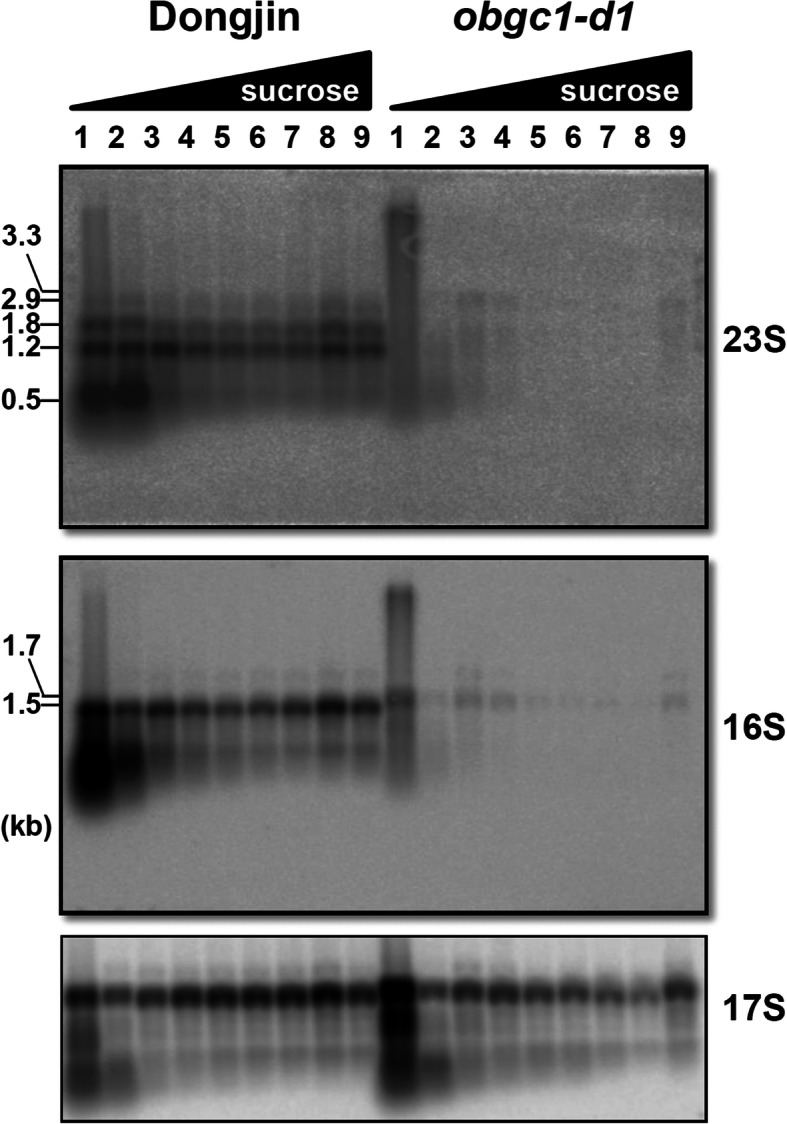
Analysis of polysome fractions in wild-type and obgc1-d1 mutant plants. Total ribosomes from leaves of 10-day-old wild-type (Dongjin) and obgc1-d1 mutant plants were separated by a sedimentation ultracentrifugation in a 15–55% sucrose gradient. Nine fractions (1–9) of equal volume were collected from top to bottom. RNAs from each fraction were separated on 1.2% agarose gels, transferred to nylon membranes, and hybridized with probes prepared from gene-specific sequences for 16S/23S rRNAs. Cytosolic 17S rRNA (17S) was used as a loading or negative control

Defective ribosome assembly strongly affects rRNA processing (Barkan 1993; Prikryl et al. 2008). To determine whether this is the case in the *obgc1-d1* mutant, we compared the distribution of plastid rRNA fragments in mutant polysome fractions with those of wild-type (Fig. 11). Leaf crude extracts from wild-type and *obgc1-d1* seedlings were fractionated by 15% to 55% sucrose density gradient ultracentrifugation and analyzed by RNA gel blot using probes specific for plastid 23S rRNAs, 16S rRNAs and cytosolic 17S rRNAs (Fig. 11). In the wild-type, the 1.8, 1.2 and 0.5 kb fragments of 23S rRNA were the major components in the polysome fractions, and the intensity of 2.9 kb bands could be clearly detected but not in a strong pattern. However, in the *obgc1-d1* mutant, polysome fractions showed only 3.3 and 2.9 kb precursors of 23S rRNA, rather than mature transcripts. In addition, the mutant showed a 1.7 kb fragment of 16S rRNA precursor, whereas the wild-type showed substantial amounts of a 1.5 kb mature transcript of 16S rRNA. It suggested that precursor ribosomes were highly accumulated as upper fractions (1–4 fractions) in the *obgc1-d*1 mutant, but not in the wild-type. The lower levels of mature 23S and 16S rRNA components in the polysomal fractions (5–9 fractions) seemed to be mainly caused by the existence of fewer polysomes in the *obgc1-d1* mutant than in the wild-type. This implies that OsObgC1 plays a fundamental role in plastid ribosome assembly, presumably through association with rRNA or specific ribosomal proteins.

## Discussion

### OsObgC1 Is a Functional AtObgC Homolog out of the Two OsObgCs in *O. sativa*

ObgC proteins are essential in chloroplast development in plants (Bang et al. 2012). Recent studies also provide a significant insight into how Arabidopsis ObgC regulates diverse physiological processes during chloroplast development, including morphological development (Bang et al. 2012), ribosome biogenesis (Bang et al. 2012), thylakoid membrane formation (Garcia et al. 2010) and environmental stress responses (Chen et al. 2014). However, the precise molecular role of *ObgC* remains unclear. The loss-function of Obg induces a lethal phenotype in most organisms, including Arabidopsis (Bang et al. 2009). Therefore, survivable *obgc1-d1* mutants in rice attracted our particular interest. Although both *OsObgC1* and *OsObgC2* expressions were in a green tissue-specific manner, only OsObgC1N-GFP, but not OsObgC2N-GFP, exhibited the punctate staining pattern, just as AtObgC-GFP did. Protein sequence alignment analysis showed that both OsObgC1 and OsObgC2 exhibited high similarity with AtObgC (71% and 37% respectively) (Bang et al. 2009). The overexpression of *OsObgC1* suppressed the embryonic lethality caused by *atobgc-t* mutation, but *OsObgC2* failed to complement *atobgc-t*, which suggests some key residues or domain which have not been explored are critical for their function or these two genes may be expressed in quite different patterns. Despite the high homology share between G domains OsObgC2 and AtObgC (Bang et al. 2009), OsObgC1 instead of OsObgC2 exhibited essential function in chloroplast development under light condition. Our data suggest that ObgC proteins are required for chloroplast genesis in monocots and dicots. However, divergent functions have been assigned to ObgC proteins in Arabidopsis and rice during evolution, as besides the corresponding OsObgC1 to AtObgC, rice possesses one more ObgC protein. Its green-tissue-specific localization implies it may be involved in chloroplast related activities. Recent study showed that OsObgC2 may be involved in the chloroplast development under cold condition which is quite different from OsObgC1 and AtObgC (Lin et al. 2018). But if OsObgC2 functions as a typical Obg protein remains unkown yet.

### ObgC Influences DNA Replication

Previous reports indicate that the activation of chloroplast genes for the transcription/translation apparatus is light-independent and occurs during a limited period in early leaf development. Besides, the expression of these genes and the chloroplast DNA content decrease once maturation of leaf cells is established (Zoschke et al. 2007; Demarsy et al. 2006). However, in this study, plastid DNA replication was continuously activated by *OsObgC1* mutation, which was reflected as a higher copy number of chloroplast DNA in the *obgc1-d1* mutant plants than those of wild-type plants. And this regulation by Obg proteins also seems conserved in *E. coli*, whose cells lacking ObgE continue to initiate replication resulting in polyploid DNA content (Foti et al. 2007).

Recently, the effects of ObgE in *E. coli* in DNA replication have been uncovered. A temperature-sensitive ObgE mutant exhibited DNA replication defect along with the decreased DnaA which is a replication initiator protein (Sikora et al. 2006). A bacterial *obgE* mutant exhibited hypersensitivity to an inhibitor HU of ribonucleotide reductase (Foti et al. 2005; Kint et al. 2012). The *obgE* mutant was significantly intoxicated due to the formation of unstructured nucleoids, which were poorly partitioned. As a consequence of the profound replication defect, chromosomes were broken and cell division was blocked. Based on these observations, it is proposed that ObgE promotes cell survival during DNA replication when replication forks are stalled (Foti et al. 2005). The depletion of *E. coli* Obg showed early chromosome-partitioning defects and accumulates replicated chromosomes, resulting in cell filamentation with polyploid DNA content. In addition, the overexpression of Obg in *E. coli* also led to aberrant chromosome partitioning, which resulted in elongated and anucleate cells (Kobayashi et al. 2001; Dutkiewicz et al. 2002). These findings suggest that these Obgs are involved directly in chromosome partitioning (Kobayashi et al. 2001), and again, that Obg is required for chromosome segregation. However, the cells with overexpression of Obg became enlarged with significantly changed distribution of chromosomal DNA, suggesting the overproduction of Obg impaired regulation of synchronization of DNA replication initiation (Dutkiewicz et al. 2002).

These enlarged nucleoids were also shown in the plastids of *obgc1-d1* mutants. It was probably caused by defects in chloroplast nucleoid division that resulted from impaired chloroplast DNA replication and partitioning by *OsObgC1* mutation. Recently, plastid ultrastructure and nucleoid morphology have been examined in Arabidopsis leaf-variegated mutant *var2*. In white sectors of *var2,* large plastid nucleoids detected by DAPI-staining were in proplastids (Sakamoto et al. 2009). It suggests that the abnormal architecture of plastid nucleoid might be indirectly caused by the arrested differentiation of proplastids into chloroplasts in *obgc1-d1* mutants.

Additionally, the *rpoA*/*Actin* ratios in both wild-type and mutant rice plants were reduced under light condition, compared to dark condition. The similar phenomenon has been also observed in maize, that cpDNA was extensively degraded during light-stimulated chloroplast development, falling to undetectable levels in many mature chloroplasts (Zheng et al. 2011). As the plastids develop, their DNAs may be damaged in oxidative environments created by photo-oxidative reactions and photosynthetic/respiratory electron transfer (Kumar et al. 2014). Although it cannot be excluded that OsObgC1 is involved in light-mediated cpDNA degradation signaling, the maintenance of higher copy numbers of cpDNA in the dark-grown mutants compared with wild-type, suggests that the function of OsObgC1 is to inhibit cpDNA replication, rather than to degrade cpDNA. The function of OsObgC1 in the chloroplast DNA replication still requires further investigation.

### ObgC Influences Ribosome Assembly in the Plastid Nucleoid

Consistent with the proposed endosymbiotic origin of chloroplasts from ancestral free-living cyanobacteria, chloroplast translation shares many of the typical features of prokaryotic protein synthesis, for example, chloroplasts possess the 70S-type ribosomes but not the 80S-type ribosomes which widely exist in most eukaryotic cells or organelles (Marin-Navarro et al. 2007). The 70S ribosome is composed of 30S and 50S subunits (Manuell et al. 2007); their biogenesis is initiated by transcription of a large pre-rRNA precursor, and they are subsequently processed, folded and assembled with r-proteins (Cheng and Deutscher 2003; Kaczanowska and Ryden-Aulin 2007). These complicated events are also catalyzed and elaborately regulated by diverse non-ribosomal factors (Kaczanowska and Ryden-Aulin 2007). It is noteworthy that among these factors, GTPases play key roles in the events (Karbstein 2007; Koller-Eichhorn et al. 2007). Bacterial Obg proteins are universally proposed with molecular function in ribosome assembly (Feng et al. 2014). One of the common features of over-expressing bacterial Obgs in *E. coli* is decreased growth rate (Polkinghorne and Vaughan 2011; Feng et al. 2014). Moreover, the region of OsObgC1 (294–752 residues) shows high similarity with ObgE and other bacterial Obgs (Chen et al. 2016). To test the effect of GST-OsObgC1_Δ1–293_ (OsObgC1 without chloroplast-transmit peptides) expression on cell growth, the serial dilution-spotting assays were performed as previously described (Feng et al. 2014). As illustrated in Supplementary Figure S2, the growth of the GST-OsObgC1_Δ1–293_ overexpressed cells was compromised compared to that of GST expressed cells under the 0.1 mM IPTG induction. We therefore hypothesized that OsObgC1 could have a similar function involved in ribosome assembly just like ObgE in bacterial cells.

In addition, we found that protein synthesis in the plastids of *obgc1-d1* mutants was severely affected as the consequence of a specific defect in the processing of the chloroplast rRNA precursor transcripts. Previously, bacterial Obgs have been reported to be associated with the 50S ribosomal subunit (Lin et al. 2004; Wout et al. 2004; Scott et al. 2000). Additionally, AtObgC was found to co-precipitate only with 23S rRNA but not with 16S rRNA, suggesting that AtObgC is associated with 50S ribosomal subunit within chloroplasts (Bang et al. 2012). It is assumed that the defect in precursor 16S rRNA processing is an indirect consequence rather than a direct one due to dysfunctional ObgC: chloroplast rRNAs in normal plants are mainly transcribed under the direction of PEP promoter, but it is reasonable to hypothesize that in *obgc1-d1* mutants precursor 16S rRNAs may be transcribed with the help of NEP promoter owing to the severe diminution of PEP activity. If this is the case, the NEP-initiated transcripts would modify the cleavable 5′ end of precursor 16S rRNA and thereby, the cleavage efficiency of the modified rRNAs may be diminished. Such a defective 16S rRNA processing in the *obgc1-d1* mutants (Fig. 9b and 10b) is also assumed to give rise to the indirect consequence of the deficiency in mature 70S particles, as previously reported (Charollais et al. 2003; Gutgsell et al. 2005; Jiang et al. 2006). Altogether, these results suggest that *obgc1-d1* mutations lead directly or indirectly to fail in plastid rRNA maturation through their accumulation and inefficient processing, which probably result in defective protein biosynthesis within the chloroplasts of *obgc1-d1*mutants.

Besides DNA or RNA related proteins, numerous ribosomal proteins and ribosome biogenesis factors have been identified in a comprehensive proteomic analysis of the maize chloroplast nucleoid. Many of these nucleoid-enriched ribosome biogenesis factors function in ribosome assembly, rRNA processing and ribosome maturation. Human ObgH1 and mitochondrial ribosomal proteins were co-purified with mitochondrial nucleoids of human cells, which suggests that ObgH1 may play a role in ribosome related events (Tschochner and Hurt 2003). ObgC has been also found in plastid nucleoid-enriched fractions from maize leaves, this finding implies that plastid ribosome assembly probably takes place in nucleoids (Majeran et al. 2012). Mitochondrial ribosomes and translation factors were co-purified with mitochondrial nucleoids of human cells, in the affinity protein purification of tagged mitochondrial DNA binding proteins (He et al. 2012). The DNA and ribosome phenotypes are linked, as in the absence of MPV17L2 proteins of the small subunit of the mitochondrial ribosome were trapped in the enlarged nucleoids (Dalla Rosa et al. 2014). These findings suggest an intimate association between nucleoids and the machinery of protein synthesis in mitochondria. However, bacterial Obgs function in ribosome biogenesis, which show cytosol localization (Scott et al. 2000; Sato et al. 2005). To confirm the possibility that OsObgC1 is involved in ribosome assembly in nucleoids, first, the ribosomal GFP-tagged proteins, *OsL13-GFP* and *OsL11-GFP* were transformed into *N. benthamiana*. Ribosomal proteins L13 and L11 also exhibited a punctate pattern in chloroplasts (Supplementary Figure S3H and L), implicating that they are nucleoid localized proteins. Further comparison of the punctate patterns between L13 and L11 revealed that one or two large dot-like signals of L13 (Supplementary Figure S3H) could be distinguished from the more numerous smaller dot-like signals of L11 (Supplementary Figure S3L). As shown in Supplementary Figure S3A, the specific pattern of OsObgC1-GFP signal proves its chloroplast nucleoid targeting. Previous studies have shown that *B. subtilis* Obg interacts with L13, instead of L11 (Scott et al. 2000). Moreover, plastid L13 is essential for embryo development in Arabidopsis, like AtObgC (Bryant et al. 2011), but not L11 (Pesaresi et al. 2006). According to the bacterial Obg function in chromosome segregation (Foti et al. 2007), the large dot signal seemed to be the aggregated cpDNA induced by overexpression of L13 and OsObgC1. Above lines of proof implies that ObgC regulates the assembly of the ribosome large subunit of plastid through L13 in nucleoids.

Thus, bacterial Obgs has been suggested to couple DNA metabolism to the translational status (Michel 2005). Rice chloroplast, ObgC1 is one of the Obg GTPase family members, a chloroplast nucleoid component involved in the maintenance and replication of cpDNA. It is plausible that OsObgC1 may couple cpDNA metabolism and chloroplast protein synthesis.

## Conclusion

The fact has been widely accepted that plant ObgCs play essential roles in chloroplast development. Previous studies have established the links of bacterial Obgs with DNA replication, rRNA processing and ribosome assembly. Our work also suggests that like bacterial Obgs, rice ObgC1 regulates chloroplast gene expression, DNA replication, rRNA processing and ribosome assembly, as well. Taken together, our study might provide a new insight into the mechanism that plant Obgs regulate chloroplast development by extending the mechanism into DNA replication and ribosome assembly in plastid nucleiods.

## Methods

### Plant Materials and Growth Conditions

The *obgc1-d* (GSNU_Ds1068) mutants were identified and selected in a *Ds* transposant rice population (Kim et al. 2004). Dongjin (wildtype), *obgc1-d1* and *obgc1-d2* were of *Oryza sativa ssp. japonica* cv. Dong Jin background.

Seeds from Arabidopsis and rice plants were sown in soil or onto MS media (pH 5.6) containing 2.1 g l^− 1^ MS salts and 2% sucrose, and grown in a controlled growth chamber (22 °C for Arabidopsis and 25 °C for rice, 70% humidity and illumination of 70–80 μmol m^− 2^ s^− 1^ white light) with 16 h light and 8 h dark.

To examine the effects of HU on rice plants, a gradient of HU concentrations (0, 2, 4, and 8 mM) was added to the MS liquid medium used to grow the seedlings for 10 days in a controlled growth chamber (25 °C, 70% humidity and illumination of 70–80 μmol m^− 2^ s^− 1^ white light) with 16 h light and 8 h dark.

### Vector Construction

For constructing *OsObgC1N-GFP* and *OsObgC2N-GFP*, the 1.2 kb of *OsObgC1* and 588 bp of *OsObgC2* N-terminus regions were cloned in-frame with the GFP gene in the pENSOTG vector, respectively.

For complementation of the Arabidopsis *atobgc-t* mutant, full-length cDNA fragments encoding OsObgC1 and OsObgC2 were amplified from rice cDNA. The PCR product was cloned between the CaMV 35S promoter and the OCS-3′ in the binary vector pFGC1008.

To determine the localizations of OsObgC1, OsL13 and OsL11 in nucleoid, the open reading frames (ORFs) of *OsObgC1, OsL13* and *OsL11* were transferred from the gateway entry clone pENTR/D-TOPO (Invitrogen) into the binary vector pMDC83 (Curtis and Grossniklaus 2003) by recombination using LR Clonase II (Invitrogen), creating OsObgC1-GFP, OsL13-GFP and OsL11-GFP fusion driven by the CaMV 35S promoter. After sequence verification, the plasmid was transformed into *Agrobacterium tumefaciens* GV3101.

### Transient Expression Assay

The *OsObgC1N-GFP* and *OsObgC2N-GFP* constructs (20 μg) were introduced into 2-week-old Arabidopsis protoplasts by polyethylene glycol-mediated transformation, as described (Bang et al. 2008). Transformed protoplasts were incubated at 22 °C in the dark. Expression of the fusion proteins was observed 2 and 3 days after transformation using an Olympus AX-70 fluorescence microscope (Olympus, Tokyo, Japan), and the images were captured with a cooled charge-coupled device camera (Olympus DP-70). Filter sets used were XF116–2 (exciter, 475AF20; dichroic, 500DRLP; emitter, 510AF23) and XF137 (exciter, 540AF30; dichroic, 570DRLP; emitter, 585ALP) (Omega, Inc., Brattleboro, VT) for green fluorescent protein and Chlorophyll autofluorescence, respectively.

The protoplasts were isolated from *Nicotiana benthamiana* transfected with *OsObgC1-GFP* constructs (Bang et al. 2009). For DAPI staining, 20 μl of transformed protoplasts were suspended in 10 μl of TAN buffer (20 mM Tris-HCl (pH 7.6), 0.5 mM EDTA (pH 7.0), 1.2 mM spermidine, 7 mM 2-mercaptoethanol, 1% (vol/vol) glutaraldehyde, and 0.4 mM PMSF), and then 10 μl of 1 μg/ml DAPI was added (Yagi et al. 2012). After incubation for 10 min, protoplasts were checked for fluorescence under an Olympus FV1000 confocal microscope. The obtained images were processed using the Adobe Photoshop software (Mountain View, CA).

### Quantitative Real-Time PCR Analyses

Total RNA was extracted from the plant materials (seedlings or leaves, as indicated in the figure legends) using TRIzol reagent (Invitrogen). Two μg of total RNA were reverse-transcribed into cDNA using Superscript III reverse transcriptase kit (Invitrogen). Real-time quantitative PCR was performed using SsoFast EvaGreen Supermix and CFX96™ Real-Time System (Bio-Rad, Hercules, CA). The cDNAs were amplified under the following cycling conditions: (1) 95 °C for 30 s, for 1 cycle; (2) 95 °C for 5 s and 55 °C for 5 s, for 40 cycles; (2) melt-curve from 65 °C to 95 °C for 5 s/step, with 0.5 °C increment. The primer sequences used for amplifications were as follows:

- For *OsObgC1*, 5′-GAAAGGGGAGAAAGGTCCAG-3′ (as a forward primer) and 5′- CAGAAGGGTGCTCTTTCCAG-3′ (as a reverse primer);
- For *OsObgC2*, 5′-GACGTTGGTCTTGTGGGACT-3′ (as a forward primer) and 5′- CCACCAAGACGACCAAGATT-3′ (as a reverse primer);
- For *OsActin*, 5′-TATGGTCAAGGCTGGGTTCG-3′ (as a forward primer) and 5′- CCATGCTCGA TGGGGTACTT-3′ (as a reverse primer);
- For *OsrpoA*, 5′-AAGCTCTTCGCAAGGCAATA-3′ (as a forward primer) and 5′- TTCGAGAGGAGGTAGCAGGA-3′ (as a reverse primer).

### Complementation Analysis

The constructs *35S-OsObgC1* and *35S-OsObgC2* were used to transform the heterozygous *atobgc-t* line, and then hygromycin-resistant T1 transformants were selected. The *atobgc-t* homozygous/heterozygous lines carrying each of the mutant constructs were further identified under hygromycin selection conditions and were confirmed via PCR analysis of the T-DNA/*AtObgC* junction as described previously (Bang et al. 2009).

### Protoplast Isolation from Rice Leaf Blades

To prepare mesophyll protoplasts, 30 leaves were harvested from 10-day-old Dongjin and *obgc1-d1* seedlings grown on an MS medium and dissected with a razor blade. The chopped leaf sample were digested in an enzyme solution [1.5% cellulase, 0.5% macerozyme, 0.1% pectolyase, 0.6 M mannitol, 10 mM MES, 1 mM CaCl_2_ and 0.1% (w/v) bovine serum albumin] for 4 h at 26 °C with gentle agitation (50–75 rpm). KMC solution (150 mM KCl, 100 mM MgCl_2_ and 100 mM CaCl_2_) was added afterward. The protoplasts were sorted from the leaf debris through a nylon mesh (20 μm), then collected by centrifuging at 1000 rpm. For 2 min and re-suspended in EP3 solution (70 mM KCl, 5 mM MgCl_2_, 0.4 M mannitol and 10 mM MES; pH 5.6) at 1 × 10^6^ protoplasts ml^− 1^ (Moon et al. 2008).

### Northern and Western Blot Analyses

For plastid rRNA profiling, northern blot analysis was carried out using total leaf RNA according to the method described (Yu et al. 2008). In agreement with previous reports (Yu et al. 2008; Bollenbach et al. 2005; Barkan 1993), the indicated rRNA-specific probes were used, and likewise, the resultant signals for various precursor/mature rRNAs were exhibited.

Total proteins were prepared from both 10-day-old wild-type and *obgc1-d1* rice leaves using TRI Reagent according to the manufacturer′s instructions, and total proteins were subjected to western blot analysis using various antibodies as described previously (Ishizaki et al. 2005). RbcL and PsbA antibodies were purchased from AgriSera (http://www.agrisera.com/); also, RpoA (Ishizaki et al. 2005) and Tic110 (Chou et al. 2006) antibodies were kindly provided by Professor Yuzuru Tozawa (Ehime University, Japan) and Professor Hsou-min Li (Academia Sinica, Taiwan), respectively.

### Polysome Analysis

Polysomes were fractionated by sucrose density gradient centrifugation as described previously (Barkan 1993) with minor modifications. Shoot tissues (~ 300 mg) were homogenized in 2.5 ml extraction buffer (200 mM Tris/HCl, pH 9, 200 mM KCl, 35 mM MgCl_2_, 25 mM EGTA, 200 mM sucrose, 2% polyoxyethylene-10-tridecyl ether, 1% Triton X-100, 1% RiboLock RNase inhibitor [Thermo Scientific, http://www.thermoscientificbio.com], and and 1× protease inhibitor cocktail [Roche, http://www.roche.com/]) and centrifuged at 15,000 g for 5 min at 4 °C. The supernatant (1.5 ml) was layered onto 10 ml of a 15–55% sucrose density gradient. Fifteen percent and 55% sucrose solution were prepared by adding 0.75 g and 2.75 g sucrose to 5 ml polysome fractionation buffer (40 mM Tris/HCl, pH 8, 20 mM KCl, 10 mM MgCl_2_), respectively. Then 55% sucrose solution was transferred to the ultracentrifuge tubes followed by adding 15% sucrose solution gently on the top of the 55% sucrose solution. Extra attention should be paid to avoid mixing these two solution layers. The 1.5 ml of supernatant was placed on the top of the 15% sucrose layer gently and was centrifuged along with the two layers of sucrose solutions in a Beckman SW41Ti rotor at 30,000 rpm for 1 h at 4 °C. During the centrifuge, the 15 ~ 55% sucrose gradient was built up. After centrifugation, 9 fractions (1.28 ml each) were collected from the top to the bottom of the gradient, and RNA was isolated. The isolated RNA was used for the Northern blot analyses of plastid rRNA as described above.

## Supplementary Information

**Additional file 1: Figure S1.** Identification of *OsObgC1 Ds* transposon insertion mutants by phenotypic and PCR method. **Figure S2.** The effect of overexpression of OsObgC1_Δ1-293_ in *E. coli* on the cell growth. **Figure S3.** OsL13-GFP and OsL11-GFP exhibited a punctate staining pattern in chloroplasts.

## Data Availability

The data sets supporting this article are included in the article and in the additional files.

## Abbreviations

At: *Arabidopsis thaliana*
cpDNA: chloroplast DNA
HU: Hydroxyurea
NEP: Nucleus-encoded polymerase
Obg: Spo0B-associated GTP-binding protein
ObgC: Chloroplast-targeting Obg GTPase
Os: *Oryza sativa*
PEP: Plastid-encoded polymerase
qRT-PCR: quantitative real-time PCR
RNR: Ribonucleotide reductase
SAM: Shoot apical meristem
