# Supplementary material for: Oryza sativa ObgC1 Acts as a Key Regulator of DNA Replication and Ribosome Biogenesis in Chloroplast Nucleoids
Source: Rice (N Y). 2021 Jul 12;14:65. doi: 10.1186/s12284-021-00498-5 (PMC8275814; doi:10.1186/s12284-021-00498-5)
Supplement: Supplementary file 1 — Additional file 1: Figure S1. Identification of OsObgC1 Ds transposon insertion mutants by phenotypic and PCR method. Figure S2. The effect of overexpression of OsObgC1Δ1-293 in E. coli on the cell growth. Figure S3. OsL13-GFP and OsL11-GFP exhibited a punctate staining pattern in chloroplasts. [file 12284_2021_498_MOESM1_ESM.docx]

**Supplementary data**

**Supplementary Fig. S1. Identification of *OsObgC1* *Ds* transposon insertion mutants by phenotypic and PCR method.**

(A) Phenotypes of *obgc1-d* mutants and wild-type plants were photographed 10 days after sowing. Scale bar = 2 cm. (B) Polymerase chain reaction (PCR) products generated by specific primers of *Ac* and *Ds* transposons were loaded on the agrose gel. In *obgc1-d1*, *OsObgC1* is knocked out by *Ds*. In *obgc1-d2*, immobile *Ds* elements are transposed by the presence of *Ac*. And as the result, knock-down mutant *obgc1-d2* was obtained with somatic transposition of *Ds* insertion.

**Supplementary Fig. S2. The effect of overexpression of OsObgC1_Δ1-293_ in *E. coli* on the cell growth.**

The *E. coli* BL21 strains with empty pGEX vector or pGEX-*OsObgC1*_Δ1-293_ were serially diluted (10^-3^, 10^-4^, 10^-5^, 10^-6^ and10^-7^) on the LB plates with 0.1 mM IPTG. Cell cultures were incubated at 37 °C for 12 h.


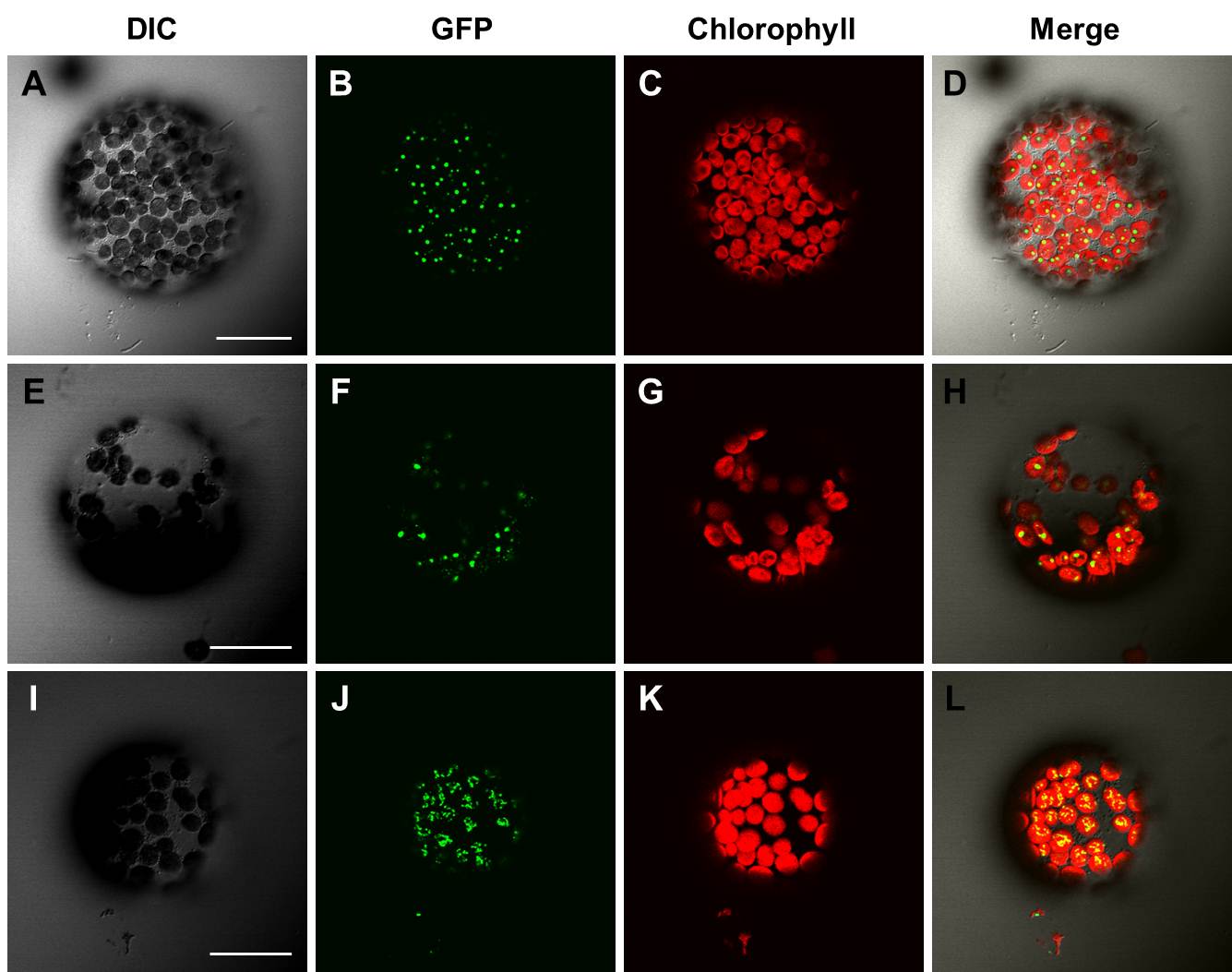


**Supplementary Fig. S3. OsL13-GFP and OsL11-GFP exhibited a punctate staining pattern in chloroplasts**

OsObgC1-GFP (A-D), OsL13-GFP (E-H) and OsL11-GFP (I-L) were observed under a confocal microscopy. DIC (differential interference contrast) and Merge (GFP + Chlorophyll) images are shown. Chlorophyll was used as a marker to detect chloroplast distribution. White bars in the DIC images represent 20 μm in length.
